# Prognostic relevance of bone marrow immune cell fractions in newly diagnosed B-cell non-Hodgkin lymphoma patients

**DOI:** 10.1080/07853890.2025.2490825

**Published:** 2025-04-15

**Authors:** Luciana Valvano, Rocchina Vilella, Fiorella D’Auria, Giovanni D’Arena, Rossana Libonati, Michela Soda, Alessia Telesca, Giuseppe Pietrantuono, Giovanna Rosaria Mansueto, Oreste Villani, Simona D’Agostino, Giovanni Calice, Teodora Statuto

**Affiliations:** a Laboratory of Clinical Research and Advanced Diagnostics, Centro di Riferimento Oncologico della Basilicata (IRCCS-CROB), Rionero in Vulture, Italy; b Laboratory of Clinical Pathology, Centro di Riferimento Oncologico della Basilicata (IRCCS-CROB), Rionero in Vulture, Italy; c Hematology, “S. Luca” Hospital, ASL Salerno, Italy; d Hematology and Stem Cell Transplantation Unit, Centro di Riferimento Oncologico della Basilicata (IRCCS-CROB), Rionero in Vulture, Italy; e Laboratory of Preclinical and Translational Research, Centro di Riferimento Oncologico della Basilicata (IRCCS-CROB), Rionero in Vulture, Italy

**Keywords:** B-cell non-Hodgkin lymphoma, DLBCL, immune cells, B cells, Th cells, double positive T-cells, prediction staging disease, prognostic role, flow cytometry

## Abstract

**Introduction:**

Non-Hodgkin lymphomas (NHLs) are the most common hematological malignancies worldwide. Among these, B-cell lymphomas (B-NHLs) are the second leading cause of death in hematologic neoplasms.

**Material and methods:**

In this study, a detailed immunophenotypic analysis of lymphocytes in the bone marrow aspirate (BMA) of 75 patients with four different subtypes of B-NHLs was performed at diagnosis. The samples were analyzed by flow cytometry (FC) using a stain-lyse-no wash technique and a comprehensive six-color antibody panel.

**Results:**

Our data showed a different trend in the percentage values of the distinct lymphocyte subsets, which did not seem to correlate with a worse prognosis, except for B cells in diffuse large B-cell lymphoma (DLBCL), which were significantly higher in stage IV than in stages II and III. ROC curve analysis showed that the B-cell percentage value could be used to predict the stage of the disease. Total lymphocytes and B cells were greater in lymphomas that presented a lower percentage of disease progression, specifically mantle cell lymphoma (MCL) and marginal zone lymphoma (MZL). In contrast, natural killer (NK) and T cells showed higher values in DLBCL and follicular lymphoma (FL), which progressed more frequently. Interestingly, in DLBCL patients with higher percentage values of double positive (DPT) and helper T cells (Th), we observed a good prognosis. Specifically, univariate Cox regression analyses indicated that a higher value of Th cells at diagnosis was a better prognostic predictor in patients with DLBCL.

**Conclusions:**

These preliminary findings encourage us to further investigate the role of lymphocyte subpopulations in B-cell NHL.

## KEY MESSAGES

1. This study provides a quick and inexpensive method for staging DLBCL by evaluating the percentage of CD19+ B lymphocytes using cytofluorimetry.
2. In addition, the study showed that the evaluation of DPT and Th subsets can provide prognostic/predictive data, supporting their role in the immune response to cancer.

## 1. Introduction

Non-Hodgkin lymphoma (NHL) is the most common type of blood cancer, with 85% of cases being B-cell malignancies [1]. B-NHLs are a heterogeneous group of cancers ranging from relatively indolent lymphomas (low-grade) to highly aggressive lymphomas (high-grade) [2,3]. They can arise at any stage of normal B cell development with distinct clinical, histopathological, and molecular characteristics. Among the aggressive forms, diffuse large B-cell lymphoma (DLBCL) is the most frequent, whereas mantle cell lymphoma (MCL) represents a unique subtype of aggressive NHL, which tends to present in older patients with a more indolent presentation at diagnosis [1]. Instead, follicular lymphoma (FL) and marginal zone lymphoma (MZL) represent the most common subtypes of indolent B-NHL (iNHL) [4]. Despite the slowing pattern and indolent behavior of iNHL, patients have been characterized by relapsing and remitting disease over many years or decades [5]. In addition, approximately 15–20% of FL patients have a more aggressive clinical course that involves early disease progression, histologic transformation, and premature death [6].

Despite recent advances in lymphoma treatment, including chemotherapy, radiotherapy, monoclonal antibodies, and hematopoietic stem cell transplantation, the clinical outcomes of patients with relapsed or refractory disease remain poor [7].

Several immunotherapeutic approaches have been developed and are being further evaluated in clinical trials, including checkpoint inhibitors, antibodies, antibody-drug conjugates, tumor vaccines, and cell-based therapies. Between 2017 and 2021, four different CD19-directed chimeric antigen receptor T-cell (CAR-T) products have been approved by the Food and Drug Administration (FDA) for their clinical use in R/R B-NHL (Axi-cel, Yescarta; Tisa-cel, Kymriah; Liso-cel, Breyanzi; Brexu-cel, Tecartus) [1,7,8]. More recently, an important breakthrough has been made with the advent of off-the-shelf bispecific antibodies [9–11]. However, there is still more to be done to define the best conditions and strategies for using these drugs to treat B-NHL, to minimize toxicity, and to identify predictive biomarkers of response and resistance [11,12].

To evade immune responses and progress, malignant cells need dynamic and mutual communication with components of the tumor microenvironment. In particular, increasing evidence has shown that immune cells are able to infiltrate tumors by promoting pro- and anti-tumorigenic functions. Thus, a deeper knowledge of the interaction between lymphoma cells and surrounding immune cells is needed to better understand the pathogenesis and prognosis of cancer, as well as new therapeutic targets [13].

In recent decades, there has been a growing interest in minor T-cell subsets, such as double negative T (DNT), double positive T (DPT), and natural killer T (NKT) cells, due to their relevant role in tumor immune surveillance. DNT cells, also called natural suppressor cells, although account only 1-5% of peripheral lymphocytes [14], play important roles in graft-versus-host-disease (GvHD), autoimmune disease, and oncotherapy [15]. They are characterized by the expression of T-cell receptor (TCR) alpha-beta (αβ) or gamma-delta (γδ) chains and CD3, but not CD4 and CD8 co-receptors [16,17].

DPT cells, identified by the co-expression of CD4 and CD8 (CD4^bright+^ CD8^dim+^ and CD4^dim+^ CD8^bright+^), display effector and central memory phenotypes [18–20]. They have been found in the peripheral blood (PB) of various species, including humans (3%), in healthy as well as pathological conditions such as viral infection, inflammatory/autoimmune disease, and cancer [21]. However, in the last decade, controversial findings regarding DPT function have been published, reporting case-specific cytotoxic or suppressive roles [21,22].

NKT cells are a very rare subset of innate-like T lymphocytes (less than 1% in human PB) [23] expressing natural killer (NK) and T cell receptors and are defined by CD1d-restricted antigen recognition [24]. Based on the TCR repertoire expression, they are classified into two different populations: “invariant natural killer T” (iNKT) and “non-invariant” NKT” cells [25]. NKTs participate in different kinds of immune responses, including antitumor immune responses through direct CD1d-dependent cytotoxicity or indirectly *via* interaction with tumor-associated macrophages (TAMs), dendritic cells (DCs), and activation of T and NK cells [24]. Several studies have documented a positive association between the number and/or functional activity of NKT cells and clinical outcomes in patients with cancer [26–30].

The assessment of immunophenotypic profile is widely recognized as useful not only for the accurate identification of the most relevant markers contributing to the detection and diagnosis of B-cell lymphoma [31–37] but also for the characterization of the lymphoma microenvironment in order to design rational immunotherapeutic strategies [38–41].

Based on these findings, the current study aimed at elucidating the interaction between immune cells and tumor cells in relation to the initiation, progression, and prognosis of B-cell lymphoma by focusing on minor T cell subsets. We specifically addressed this issue by analyzing the bone marrow aspirate (BMA) of 75 patients affected by DLBCL, FL, MCL, and MZL through FC. Given the retrospective nature of the study and the inability to assess CD1d and/or V-alpha-24 receptor expression, rather than NKT, we analyzed an NKT-like subpopulation, phenotypically identified by co-expression of TCR and NK-associated receptors, such as CD56 and CD16 [42].

## 2. Materials and methods

### 2.1. Patients population

This is a monocentric study with retrospective collection of biological and clinical data, conducted according to the principles of the Declaration of Helsinki and approved by the Comitato Etico Unico Regionale per la Basilicata (approval number 20200034750). Patients who met the eligibility criteria (new B-NHL diagnosis, psychomotor integrity, and age > 18) were enrolled after obtaining written informed consent for the use of biological samples for research purposes.

### 2.2. Flow cytometric analysis

Following clinical practice guidelines, we routinely collected about 2-3 mL of BMAs into an EDTA tube within 1 month of diagnosis for FC analysis. A stain-lyse-no wash technique and a comprehensive six-color antibody panel (FITC/PE/PerCP-Cy5-5/PE-Cy7/APC/APC-H7 fluorescent conjugates) on a BD FACS Canto II (BD Biosciences, BD, Franklin Lakes, NJ, USA) or “Navios 10/3” (Beckman Coulter, BC, Brea, CA, USA) were used.

Briefly, fluorescently labeled antibodies (BD or BC) were mixed with 100 μl of BMA and incubated for 15 min in the dark at room temperature. Subsequently, the sample was lysed with the lysing solution according to the manufacturer’s instructions. Stained cells were analyzed with a flow cytometer and data were analyzed using Kaluza software version 2.1 (BC).

The "rare event" visualization function has been used to display small subpopulations, identified by specific markers from about 15,000 events in the lymphocyte gate. The percentage value of lymphocytes was given to the total leukocytes, whereas the percentage value of each lymphocyte subpopulation was calculated starting from the lymphocyte gate. The gating strategy was previously described in our recent study [43]. In summary, B cells were defined as CD19^+^, total T cells as CD3^+^, helper T (Th) cells as CD3^+^ CD4^+^, cytotoxic T cells (Tc) as CD3^+^ CD8^+^, DNT cells as CD3^+^ CD4^−^ CD8^−^ CD16/CD56^−^, DPT cells as CD3^+^ CD4^+^ CD8^+^, NKT-like cells as CD3^+^ CD16/CD56^+^, and NK cells as CD3^−^ CD16/CD56^+^.

### 2.3. Statistics

Statistical analyses were performed using R software [44] and the IBM SPSS statistical package (International Business Machines Corporation, Armonk, NY, USA, version 28). All graphs were generated using GraphPad Prism V.8.0.1 (GraphPad Software). Continuous variables were expressed as medians and IQR. Most data series were found to deviate from a normal distribution (Kolmogorov–Smirnov test). Thus, comparisons among groups were performed using the Kruskal-Wallis test and two-sided Mann–Whitney *U* test. Spearman’s correlation test was used to evaluate the possible correlation of the data series. Receiver operating characteristic (ROC) curves and the area under the curve (AUC) were used to analyze the relevance of lymphocyte subsets in predicting disease stage. Using the Kaplan–Meier method, overall survival (OS) and progression-free survival (PFS) were estimated, and differences between groups were evaluated using the log-rank test. OS was defined as the interval from diagnosis to death from any cause or the last follow-up; PFS was defined as the interval from the start of treatment to the evidence of progressive disease, death, or the last date the patient was known to be progression-free or alive. Cox proportional hazards regression models were fitted to assess the associations between patient characteristics and time-dependent variables.

Differences were considered statistically significant at *p* < 0.05.

## 3. Results

### 3.1. Patient characteristics

This retrospective study involved 75 patients (median age 66, range 31–86) with four different subtypes of B-NHL (40 DLBCL, 5 FL, 10 MCL, and 20 MZL) [45,46] admitted at Referral Cancer Center of Basilicata IRCCS-CROB from June 2011 to December 2018. Tumor stage was scored using the Cotswolds-modified Ann Arbor classification [47]. The main clinical and biological features of patients are reported in Table 1.

**Table 1.** Clinical–biological features of patients with B-NHL.

Clinical-biological features		Age, M (R)^a^	Males, N (%)^a^	Pts with MCC, N (%)^a^	WBC count (µL^−1^), M (IQR)^a^	Treatment, N treated patients (%)^a^	CR, N (%)^a^ †, N (%)	PR, N (%)^a^ †, N (%)	R, N (%)^a^ †, N (%)		OS mths, Mdn (IQR)^1^	PFS mths, Mdn (IQR)^1^	Cells Mdn, % (IQR)^4,a^	
										PD, N (%)^a^†, N (%)			Ly B NK T	Tc Th DNT DPT NKT-like
N. Patients: 75		66 (31–86)^1^	46 (61.3)^1^	34 (45.3)^1^	7305 (4300)^1^	74 (98.7)^1^	51 (69.9)^2^	15 (20.6)^2^	12 (16.9)^2^	5 (7.1)^2^	36 (30)	29 (31.5)		
subtypes N (%)^1^	stage, N (%)^a^													
DLBCL 40 (53.3)	IIA/B, 11 (27.5) IIIA/B, 10 (25) IVA/B, 18 (45) n.a., 1 (2.5)	67.5 (31–86)	24 (60)	14 (18.6)	7480 (4510)	- R-Bendamustine, 2 (5) - R-CHOP, 11 (27.5) - R-CODOX-M/R-IVAC, 5 (12.5) - R-COMP, 17 (42.5) - R-CVP, 2 (5) - R-DHAP+ FEAM, 1 (2.5) - R-GemOx, 1 (2.5) - R-MACOP-B, 1 (2.5)	28 (70)	7 (17.5)	6 (15)	3 (7.5)	32.5 (43)	28 (35)	13.19 (13.24) 8.76 (8.96) 9.39 (11.40) 77.19 (17.10)	32.94 (16.82) 37.00 (19.78) 1.82 (2.04) 1.06 (1.47) 8.93 (8.34)
							2 (5)	3 (7.5)	6 (15)	2 (5)				
FL 5 (6.6)	IIIA/B, 4 (80) IVB, 1 (20)	64 (44–85)	4 (80)	2 (2.7)	7630 (4135)	- R-Bendamustine, 1 (20) - R-CHOP, 3 (60) - R-CVP, 1 (20)	3 (60) 0 (0)	1 (20) 0 (0)	1 (20) 0 (0)	1 (20) 1 (20)	43 (44)	32 (36)	12.61 (5.89) 9.33 (9.07) 16.55 (18.19) 71.45 (20.92)	25.64 (16.04) 34.90 (14.56) 3.65 (3.91) 0.67 (0.74) 4.73 (7.99)
MCL 10 (13.3)	IIA, 1 (10) IIIA, 3 (30) IVA/B, 6 (60)	69.5 (55–84)	8 (80)	6 (8)	6950 (2170)	- Hyper-CVAD-R,1 (10) - Ibrutinib, 2 (20) - R-BAC, 4 (40)^c^ - R-Bendamustine, 1 (10) - R-CHOP, 1 (10) - Hyper-CVAD, 1 (10)	8 (80) 2 (20)	1 (10) 0 (0)	3 (30) 1 (10)	1 (10) 1 (10)	36 (18)	34.5 (20)	24.23 (53.20) 21.61 (72.21) 7.72 (19.60) 56.33 (62.63)	27.16 (34.66) 17.53 (23.86) 1.70 (1.76) 0.41 (0.4) 4.19 (6.52)
MZL 20 (26.7)	IIA, 3 (15) IIIA, 7 (35) IVA/B, 10 (50)	64,5 (40–83)	10 (50)	12 (16)	5940 (5370)	- Rituximab, 5 (25) - R-Bendamustine, 3 (15) - R-CVP, 10 (50) - R-IEV,1 (5) - n.a., 1 (5)	12 (63)^3^ 2 (10.5)^3^	6 (31.6)^3^ 2 (10)^3^	2 (10.5)^3^ 0 (0)^3^	0 (0)^3^ 0 (0)^3^	38 (30)	15 (34.5)	24.48 (21.48) 27.13 (45.42) 6.94 (10.23) 62.40 (33.32)	22.50 (17.63) 30.56 (16.68) 1.57 (1.23) 0.49 (0.50) 4.05 (5.39

The median (Mdn) and Interquartile Range (IQR) of the percentage values are reported.^1^ Data available for 75 patients,^2^ data available for 74 patients,^3^ data available for 19 patients^4^. The percentage value of lymphocytes was given on the total leukocytes, while the percentage value of each lymphocyte subpopulation was calculated starting from the lymphocyte gate. ^a^Data relating to the number of patients in each B-NHL group. †, dead patients. WBC: white blood count; Ly: lymphocytes; CR: complete response; PR: partial response; R: relapse; PD: progression disease; OS: overall survival; PFS: progression-free survival; M: mean; MCC: Major Complication and Comorbidity; mths: months; n.a.: not assigned; R-CHOP: rituximab, cyclophosphamide, doxorubicin, vincristine, and prednisone; R-CODOX-M/R-IVAC: rituximab-cyclophosphamide, vincristine, doxorubicin, high-dose methotrexate/rituximab-ifosfamide, etoposide, high-dose cytarabine; R-COMP: rituximab, cyclophosphamide, non-PEGylated liposomal doxorubicin, vincristine, and prednisone; Hyper-CVAD-R: hyperfractionated cyclophosphamide, vincristine, doxorubicin, dexamethasone – rituximab; R-BAC: rituximab-bendamustine and cytarabine; R-CVP: rituximab-cyclophosphamide, vincristine, prednisone; R-DHAP+ FEAM: rituximab-dexamethasone, cytarabine, cisplatin + fotemustine, etoposide, ARA-C, and melphalan; R-GemOx: rituximab-gemcitabine, and oxaliplatin; R-MACOP-B: rituximab-methotrexate, leucovorin (LV), doxorubicin, cyclophosphamide, vincristine, prednisone-bleomycin; R-IEV: rituximab-ifosfamide, epirubicin and etoposide.

### 3.2. Analysis of BMA lymphocyte subpopulations in B-NHL patients

For each B-NHL subtype, the percentage value of the following immune cell subpopulations was evaluated: B, T, NK, Tc, Th, DNT, DPT, and NKT-like cells.

**Figure 1.**
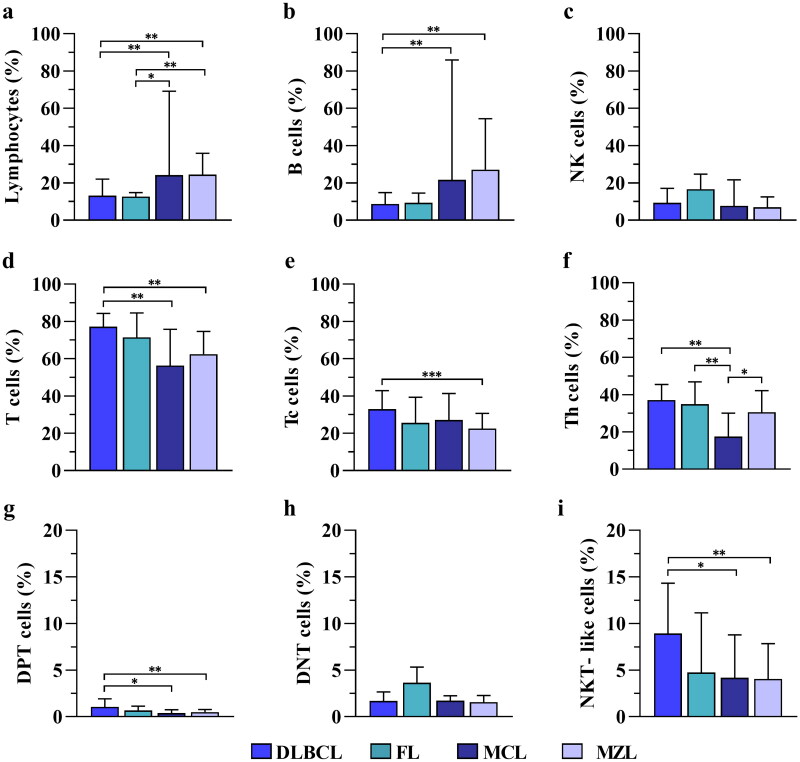
Analysis of the immune cell subpopulations in B-NHL. The histograms show the median ± IQR of: (**a**) total lymphocytes; (**b**) B cells; (**c**) NK cells; (**d**) total T cells; (**e**) Tc cells; (**f**) Th cells; (**g**) DPT cells; (**h**) DNT cells, and (**i**) NKT-like cells measured in DLBCL (*n* = 40), FL (*n* = 5), MCL (*n* = 10), and MZL (*n* = 20) patients. *p < 0.05; **p < 0.01; *** *p* < 0.001 (Kruskal–Wallis test followed by the Mann–Whitney *U* test).

Comparing each subpopulation, we documented significant variation between B-NHL subtypes, except for NK and DNT cells, both of which increased only in FL and DLBCL (Figure 1(a–i)).

In detail, the Mann-Whitney U test highlighted a significant increase in the total lymphocyte percentages in the MCL and MZL groups compared to DLBCL and FL (Figure 1(a)). A decrease in B cell value was observed in the DLBCL and FL groups, reaching statistical significance only in DLBCL compared to MCL and MZL (Figure 1(b)). Conversely, a greater percentage of total T cells (Figure 1(d)), DPT cells (Figure 1(g)), and NKT-like cells (Figure 1(i)) were observed in DLBCL patients, that were statistically significant compared to MCL and MZL. In the DLBCL group, a higher value of Tc cells was also observed, which was significant only compared to the MZL values (Figure 1(e)). In contrast, Th cell percentage was significantly lower in the MCL group than in the other lymphoma subtypes (Figure 1(f)).

### 3.3. Immune cell subtypes and B-NHL staging

**Figure 2.**
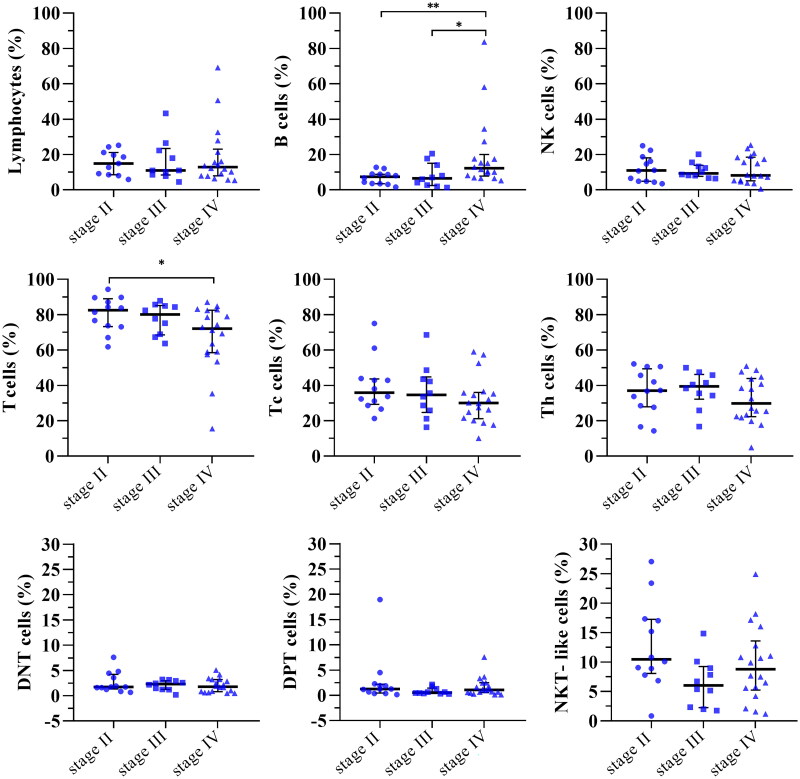
Lymphocyte subsets and staging in DLBCL patients. The scatter plots showed the individual percentage value of each lymphocyte subpopulation examined in DLBCL stage II, stage III, and stage IV patients. *p < 0.05; **p < 0.01 (Kruskal-Wallis test followed by the Mann–Whitney *U* test).

For each B-NHL analyzed, we evaluated the distribution of lymphocyte subsets in stages II, III, and IV. Specifically, only in DLBCL patients, we documented a significant increase in B cell percentage in the advanced stage (stage IV) compared with stages II and III and a significant reduction of total T cell value between stages II and IV. The remaining populations did not appear to change significantly in relation to disease (Figure 2).

**Figure 3.**
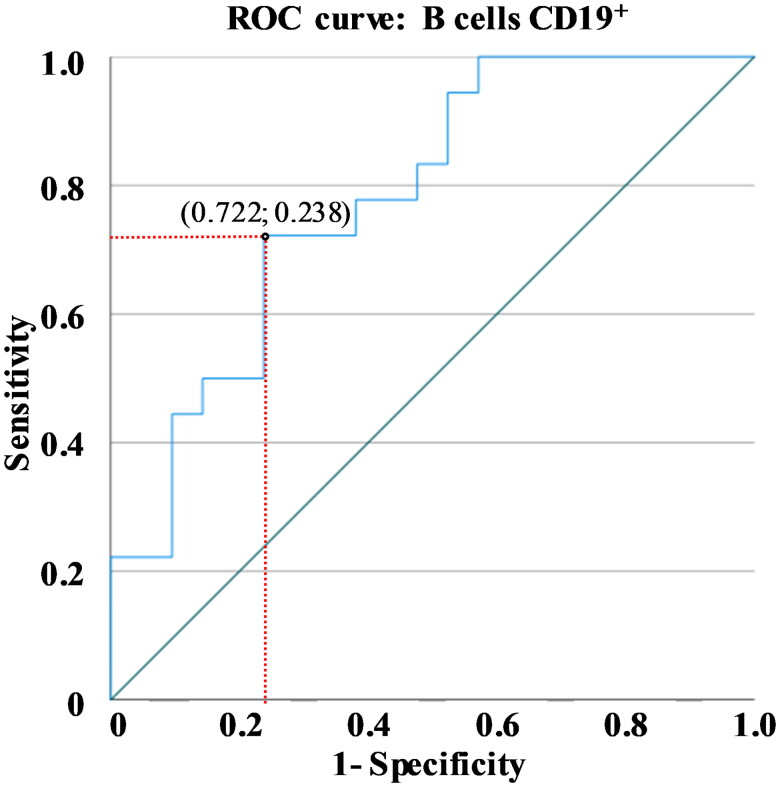
ROC curve analysis for the optimal cut-off values of B cells in DLBCL. The best cut-off coordinate (Youden’s index) for the percentage value of B cells is reported.

Data regarding the other B-NHLs are shown in the supplementary figures (Figure 1S–3S).

Interestingly, ROC curve analysis showed that the percentage value of B cells evaluated at diagnosis could distinguish DLBCL patients in stage IV and stage II/III with 72% sensitivity and 24% specificity (AUC = 0.780). The optimal cutoff value for the percentage of B cells was 9.32% (Figure 3). The analysis was also performed among the B-NHLs studied for each prognostic stage (II, III, and IV). Table 2 reports these significant results.

**Table 2.** Comparison of lymphocyte subpopulations and staging among B-NHLs.

		DLBCL	MCL		MZL	
	Lymphocyte subsets	Median (%)	Median (%)	*p*-value	Median (%)	*p*-value
Stage II	B cells	6.83	Not evaluable		14.90	0.03*
Stage III	B cells	6.54	83.60	0.03*	36.81	0.03*
	T cells	80.08	13.60	0.01*	42.00	0.01*
	Tc cells	34.59	7.29	0.12	12.61	0.01*
Stage IV	Lymphocytes	12.93	24.23	0.02*	28.33	0.05*
	DPT cells	1.06	0.33	0.02*	0.43	0.03*

*vs DLBCL.

### 3.4. Immune cell subtypes and clinical outcomes

**Figure 4.**
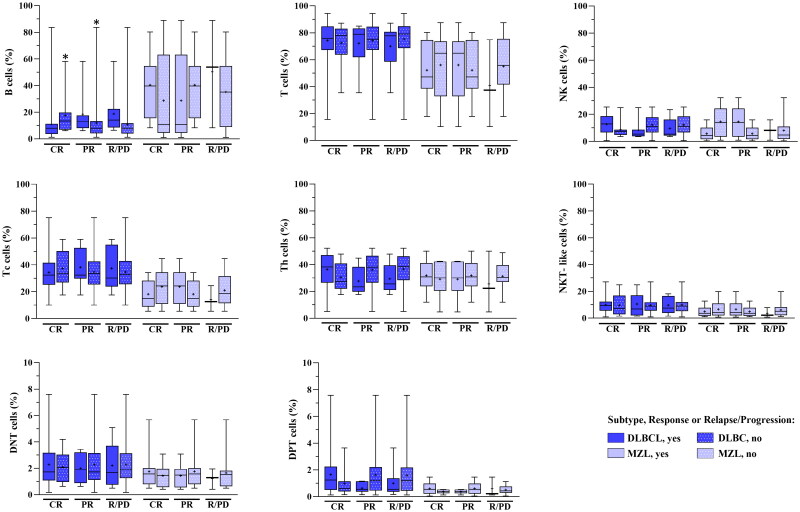
Impact of lymphocyte subtypes percentage value on therapeutic response. The box plots show the percentage values of the lymphocyte subpopulations in relationship with the different responses to the therapy in DLBCL and MZL groups. *p < 0.05 (Mann–Whitney *U* test). CR: complete response; PR: partial response; R/PD: Relapse/progression disease.

To determine the correlation between lymphocyte subsets and clinical outcomes in patients with B-NHL, we evaluated the percentage values for each subpopulation stratified by therapeutic response: complete response (CR), partial response (PR), and relapse/progression disease (R/PD). Because of the low number of cases affected by FL and MCL, which could limit the statistical analysis, we reported only the results for the DLBCL and MZL groups. Significant findings were observed in DLBCL for the percentage values of B cells, which were increased in patients with disease progression and/or those who did not achieve a complete response (Figure 4).

Although not statistically significant, the DPT cell percentage was approximately halved in DLBCL patients with PR and/or R/PD, while it doubled in those who later achieved CR. In the MZL group, we observed a DPT halving only in patients who experienced progressive disease, but the percentage was lower in patients who later had a worse prognosis (Figure 4).

**Figure 5.**
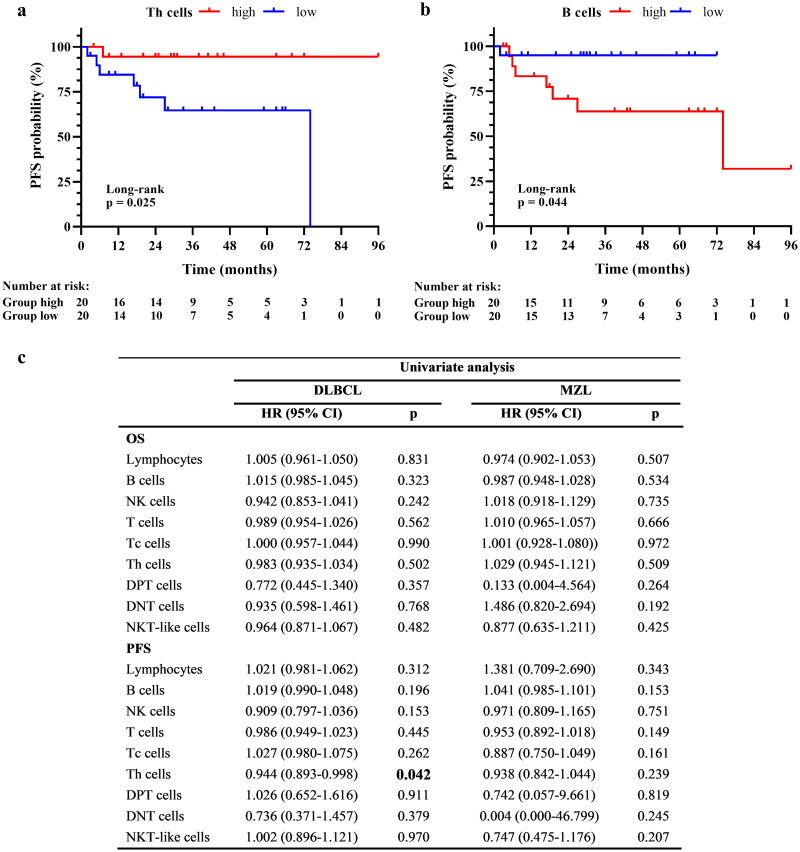
Investigation of prognostic in DLBCL and MZL. Kaplan–Meier survival curves illustrate the prognostic effect on PFS of Th (**a**) and B (**b**) cells percentage in DLBCL group. The number at risk is reported at the bottom of each curve. Cox’s proportional hazards model analysis performed in DLBCL and MZL (**c**). Continue variables: Lymphocyte cells, B cells, NK cells, T cells, Tc cells, Th cells, DPT cells, DNT cells, NKT-like cells. Statistical results with *p* < 0.05 are bolded.

According to the median percentage value of the lymphocyte subsets, DLBCL and MZL patients were divided into two subgroups: high and low. Among the cohort of 59 patients (40 DLBCL and 19 MZL), only in DLBCL group Kaplan–Meier curves documented a longer 6-year PFS in patients with a high percentage of Th cells at diagnosis (Figure 5(a)); conversely, patients with a higher percentage of B cells had a worse outcome (Figure 5(b)).

Cox regression analysis identified CD4 T cells as a better prognostic predictor in DLBCL patients at diagnosis; however, we did not show any statistically significant impact on overall survival (Figure 5(c)).

### 3.5. Th, total T cells, and DPT: correlations

Given the prognostic role of Th cells and the increase in DPT cell value observed in DLBCL patients with CR, we aimed to investigate the possible correlation of these populations with the total T cells that were increased in this subtype of lymphoma compared with MZL.

**Figure 6.**
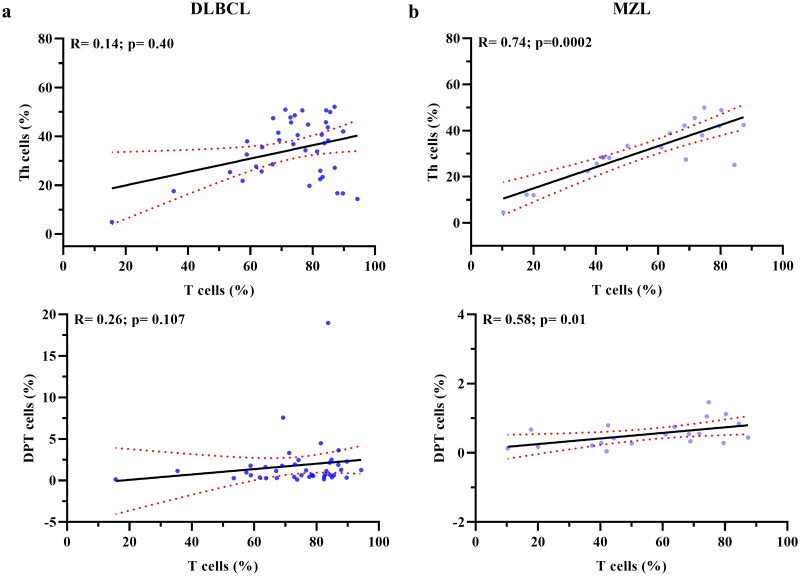
Correlation between CD3, CD4 and DPT in DLBCL and MZL patients. Spearman correlation coefficient analysis between total T cells, Th and DPT cells in DLBCL (a) and MZL (b).

The analysis did not show significant correlations in the DLBCL group (Figure 6(a)). Conversely, a significant correlation between total T vs. Th and total T vs. DPT cells was observed in MZL (Figure 6(b)). Moreover, total T cells were also significantly correlated with the remaining T subpopulations (DNT, NKT-like, and Tc) in both DLBCL and MZL (Supplementary Figure 4S).

## 4. Discussion and conclusion

The combination of immunotherapy and chemotherapy has recently emerged as a powerful solution to reduce the negative side effects of chemotherapy. The mechanisms of interaction between malignant cells and the immune system, as well as between distinct intra-tumoral subsets of the same cells, are the basis for current and future cancer immunotherapy. Immune cells have been suggested to play an essential role in these interactions with both innate and adaptive immune cells (T cells and B cells) that contribute to tumor formation and metastasis when present in the tumor microenvironment [48,49]. Cell death, specifically after chemotherapy, activates tumor-related immunity [49,50]. Indeed, immune cell infiltration into the microenvironment has been documented in a variety of malignancies, and its positive prognostic importance has been well established. Multicolor FC is a reliable and simple technology for monitoring immune cell phenotyping and routinely tracking immunological state.

In order to elucidate a putative immune activation following disease, we performed a retrospective immunological profiling using highly sensitive multiparametric FC on a longitudinal cohort of NHLs. In this study, we aimed to analyze changes in the percentage values of lymphocyte subpopulations in patients with four types of B-NHLs. To this end, eight different lymphocyte subtypes in the BMA were explored using the six-color FC technique. This study showed a different trend in the percentage values of the cellular subpopulations in the four types of lymphomas analyzed. Total lymphocytes and B cells were greater in MCL and MZL, which presented a lower percentage of disease progression. In contrast, NK and T cells showed higher values in DLBCL and FL that progressed more frequently. Specifically, only NK and DNT cells did not show any significant differences among the four lymphomas analyzed. In both cases, contrary to the trend of the remaining T populations, the highest percentages were observed in FL. However, the small number of patients in the FL group did not provide clear and significant results, placing some limitations in this study. The only statistically significant result related to FL was found for the percentages of Th lymphocytes, which were significantly increased compared to MCL.

Our recent study on B-cell chronic lymphocytic leukemia (B-CLL) supports the concept that active B-CLL immune surveillance of DNT cells is independent of disease staging [43]. In contrast, DPT and NKT-like cells appear to increase significantly only as the disease progresses. In this study, DPT cells showed a significant change in stage IV between DLBCL, MCL, and MZL. Within the same pathology, there were no differences in the percentage values in relation to prognostic stages. The only subpopulations that varied significantly with the prognostic stages were T and B cells in DLBCL. In detail, B lymphocytes were significantly higher only in DLBCL in stage IV than in stage II and III, which had similar values. This finding suggests that the BM percentage of B-cells evaluated by FC can be used as a predictive staging factor in DLBCL. In support of our hypothesis, other studies in the field of oncohematology identify the correlation between the count of CD19^+^ cells in the bone marrow and the stage of the disease [51–52].

Using ROC analysis, we identified a hypothetical B-cell cutoff: if the CD19^+^ percentage on the total lymphocytes was ≥9.32%, we had a 72% probability of correctly identifying stage IV compared to stages II/III.

Moreover, in DLBCL, higher percentage values of B lymphocytes at diagnosis are associated with a failed CR and/or R/PD, leading to a worse prognosis. Contrary to B cells, a high percentage of Th cells appear to be a favorable prognostic/predictive factor in DLBCL.

According to a recent study, there was a selective increase in lymphocytes in DLBCL patients, particularly in CD4^+^ T and NK cells, which may improve the therapeutic effect and prognosis [53]. Kusano et al. examined CD4^+^ and CD8^+^ T-cell counts in the PB of individuals with DLBCL and found a negative correlation between a low absolute number of CD4^+^ T cells at diagnosis and 5-year disease-free survival (DFS) [54].

In our study, it’s interesting to note that while the Th trend in DLBCL and MZL was comparable, in DLBCL it was unrelated to the total T lymphocyte trend, which was associated in MZL. Within the limits of significance, a similar trend for DPTs was observed in patients with DLBCL. These observations suggested that Th and DPT cells play a role in the immune response to DLBCL.

However, it must be acknowledged that these results should be interpreted within the context of their limitations. First, the different number of patients between the B-NHLs and/or prognostic groups did not provide clear statistical results. Second, we did not provide any control data due to the retrospective nature of the study and the difficulty of recruiting BMAs of healthy controls.

Moreover, by systemic flow cytometric monitoring of the immune profile of patients with DLBCL, it is possible to rapidly gain a better understanding about the disease onset and development, setting the stage for developing innovative treatment strategies and improving clinical prognosis. Specifically, DLBCL data of this study suggest that the assessment at diagnosis could influence treatment decisions or prognostic evaluations in clinical settings. CD19^+^ and Th could offer quick indications for disease staging and prognosis, prompting the clinician to accelerate the diagnostic process (e.g. histological analysis, medical imaging tests) and intensify the patient monitoring (e.g. close follow-ups) in order to early identify low/non-responder patients and a possible treatment switch or intensification, in an effort to maximize their PFS and OS.

This study assessed the non-malignant host immune system’s prognostic significance, highlighting the significance of assessing these cells’ function in immune responses against cancer. To find out how the host’s immune system and the neoplastic clone interact to affect clinical outcomes, more research is required.

An ongoing PB cells immunophenotyping study, including absolute values and expanded patient casuistry, is being conducted to confirm our findings, highlighting the potential of MFC in immune function assessment in the clinic, starting from a simple blood sample.

## Supplementary Material

Supplemental Material

## Data Availability

The data that support the findings of this study are available from the corresponding author [L.V. and R.V.] upon reasonable request.
