# Supplementary figures and images for: Prognostic relevance of bone marrow immune cell fractions in newly diagnosed B-cell non-Hodgkin lymphoma patients
Source: Ann Med. 2025 Apr 15;57(1):2490825. doi: 10.1080/07853890.2025.2490825 (PMC12001853; doi:10.1080/07853890.2025.2490825)

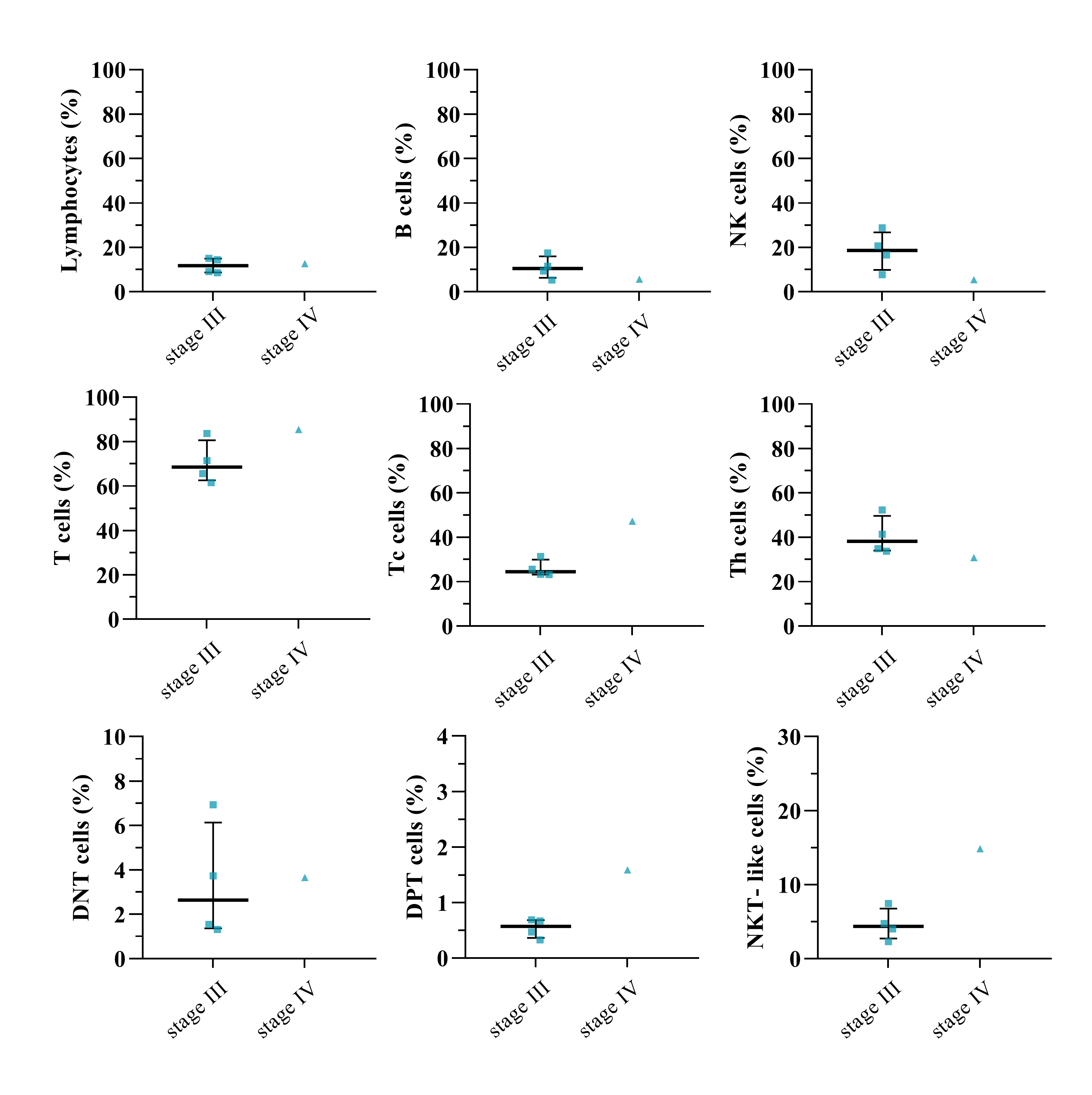

Supplement: Supplemental Material [file IANN_A_2490825_SM1959.zip › Suppl_/Figure 1S.tif]

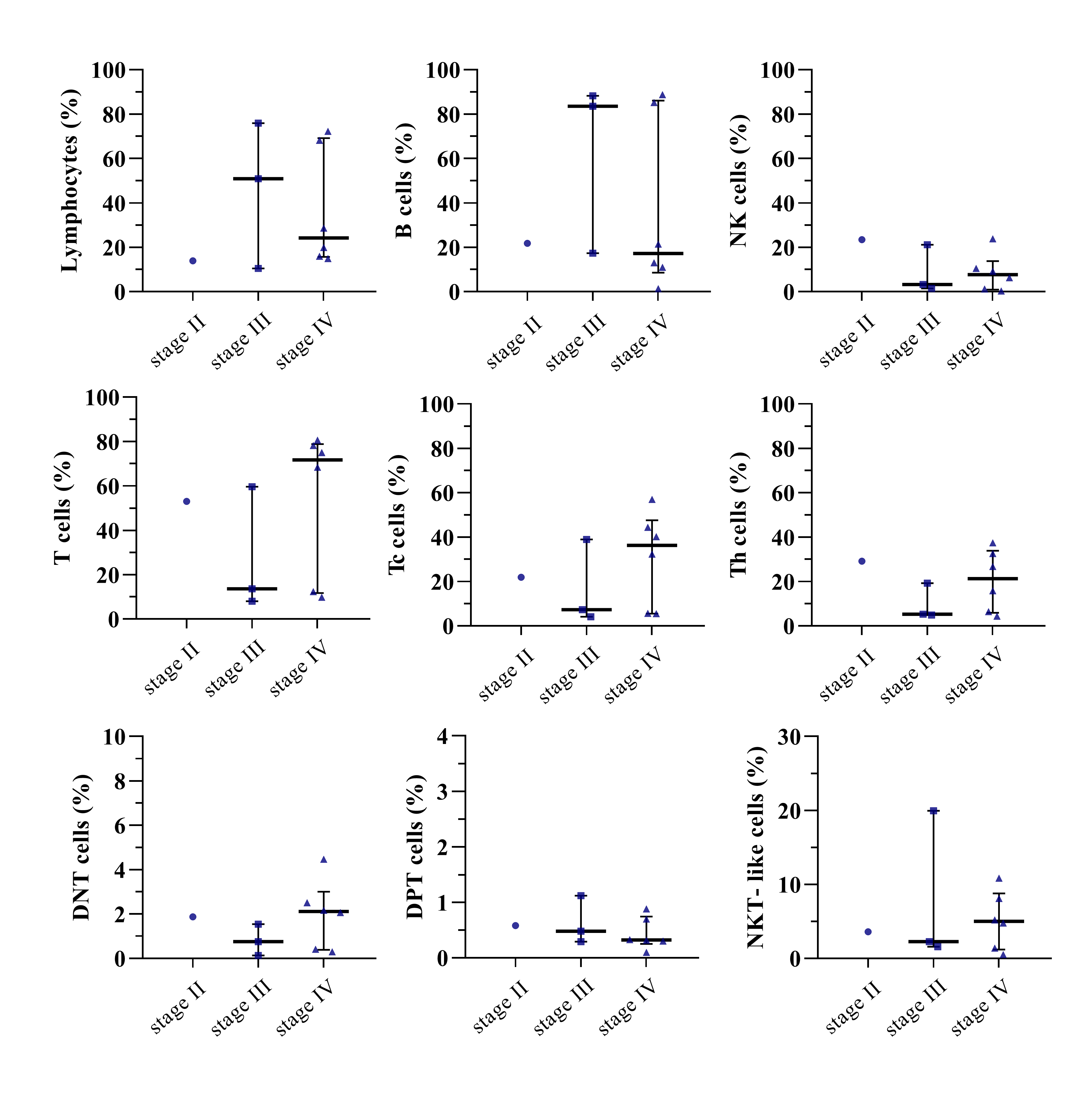

Supplement: Supplemental Material [file IANN_A_2490825_SM1959.zip › Suppl_/Figure 2S.tif]

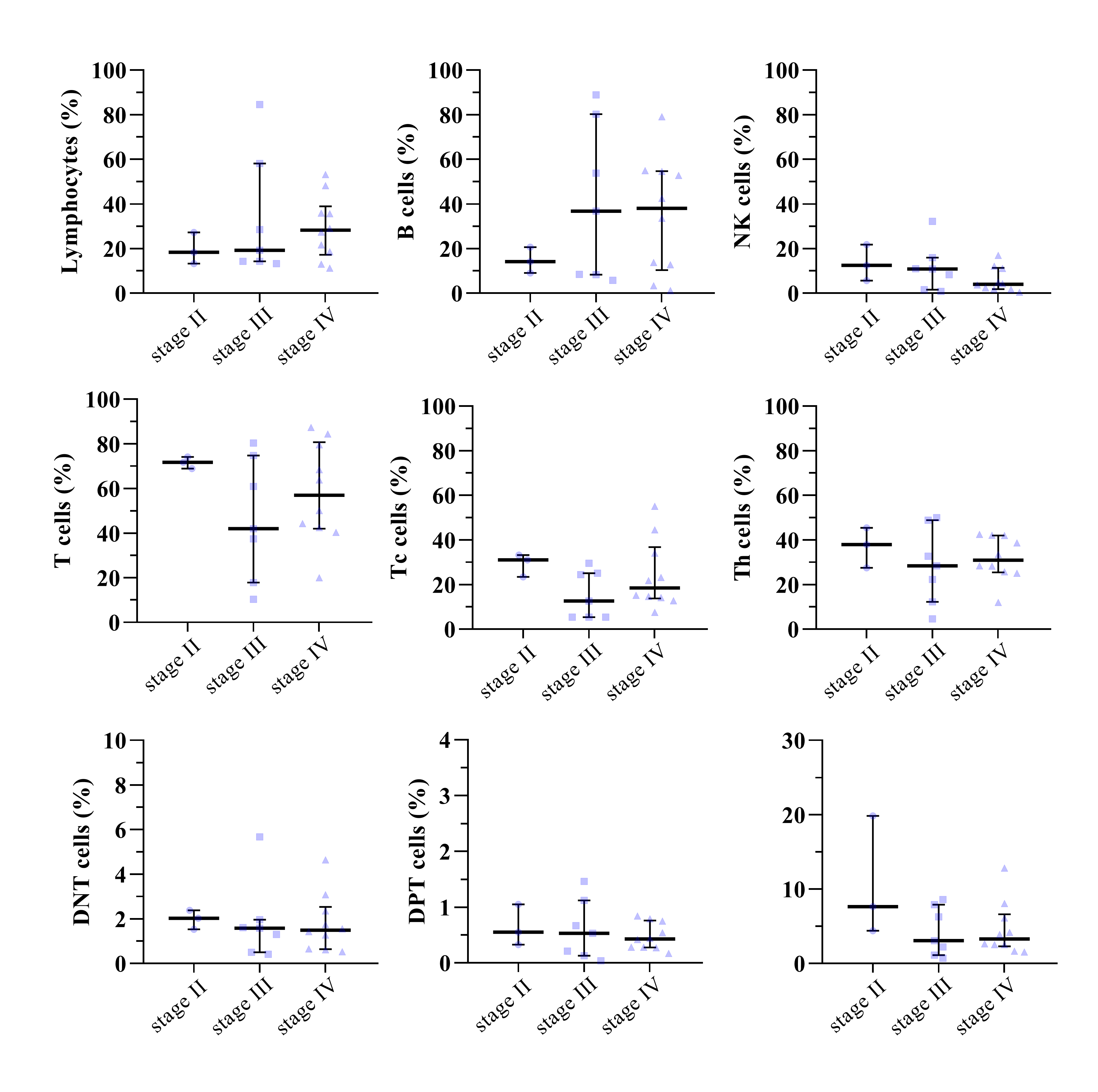

Supplement: Supplemental Material [file IANN_A_2490825_SM1959.zip › Suppl_/Figure 3S.tif]

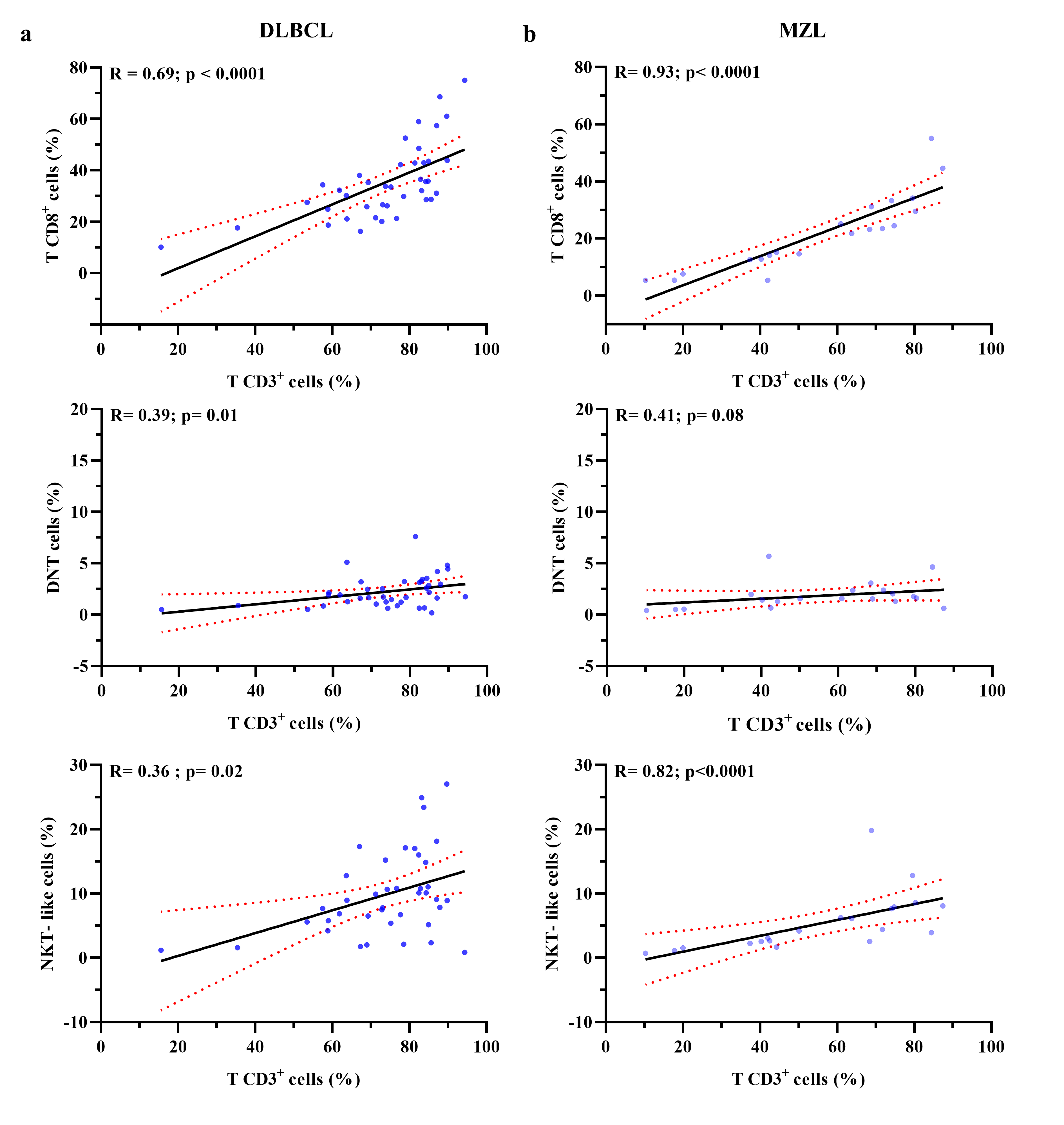

Supplement: Supplemental Material [file IANN_A_2490825_SM1959.zip › Suppl_/FIgure 4S.tif]
